# *Plasmodium vivax* circumsporozoite surface protein (*Pv*CSP) as a vaccine candidate: a brief review

**DOI:** 10.1590/S1678-9946202567066

**Published:** 2025-10-03

**Authors:** Kessia Caroline Souza Alves, Victor Calebe Alves da Costa, Emmily Myrella Vasconcelos Mourão, Joyce Marinho Melo, Maria Edilene Martins Almeida, Juliana Gomes de Souza Oliveira, Luís André Morais Mariúba

**Affiliations:** 1 FIOCRUZ Amazônia, Instituto Leônidas e Maria Deane, Manaus, Amazonas, Brazil; 2 Fundação Oswaldo Cruz, Rio de Janeiro, Rio de Janeiro, Brazil; 3 Fiocruz Amazônia, Instituto Leônidas e Maria Deane, Programa de Pós-Graduação em Biologia da Interação Patógeno Hospedeiro, Manaus, Amazonas, Brazil; 4 Fundação Hospitalar de Hematologia e Hemoterapia do Amazonas, Programa de Pós-Graduação em Hematologia, Manaus, Amazonas, Brazil; 5 Universidade Federal do Amazonas, Programa de Pós-Graduação em Biotecnologia, Manaus, Amazonas, Brazil; 6 Universidade Federal do Amazonas, Programa de Pós-Graduação em Imunologia Básica e Aplicada, Manaus, Amazonas, Brazil

**Keywords:** Circumsporozoite surface protein, CSP, Plasmodium vivax, Malaria, Vaccine

## Abstract

For decades, research and development toward malaria vaccine has prioritized infections caused by *Plasmodium falciparum*, since it is the species responsible for the most severe cases of malaria, which results in a comparatively smaller number of studies devoted to *Plasmodium vivax*. This disparity is due to complexities inherent in the biology of *P. vivax*, including the formation of hypnozoites in the pre-erythrocytic phase, difficulties in establishing robust *in vitro* culture systems, and the absence of fully representative experimental models. Among the antigens investigated for vaccine formulations, the *Plasmodium vivax* circumsporozoite surface protein (*Pv*CSP) stands out for its immunogenic potential and the partial protection observed in preclinical studies. However, clinical trials with *Pv*CSP-based vaccines have not yet demonstrated a consistent protective response. This review aims to show a historical overview of the studies that have evaluated the potential of vaccines based on the *Pv*CSP protein, as well as to explore the innovative approaches that have been investigated in the last decade to overcome the challenges and advance the development of an effective vaccine against malaria caused by *P. vivax*.

## INTRODUCTION

Malaria remains a significant public health problem in tropical and subtropical regions. In 2023, data from the World Health Organization (WHO) reported approximately 263 million cases in countries where the disease is endemic, and around 9.2 million were attributed to *Plasmodium vivax*, underlining its continued contribution to the global burden of disease^1^. *Plasmodium falciparum* is responsible for most severe and fatal cases of malaria, and scientific efforts have historically focused on this species; however *P. vivax* is the most geographically widespread human malaria parasite, with substantial challenges for its elimination^2,3^.

In Brazil, about 142,000 malaria cases were reported in 2023, of which about 140,000 occurred in the Brazilian part of the Amazon. *P. vivax* was the predominant species among autochthonous cases in the country, accounting for around 117,000 cases, and 115,000 were concentrated in the Amazon region of the country, highlighting its epidemiological relevance in Brazil^4^.

*P. vivax* infection affects individuals of all age groups, manifesting itself from mild clinical pictures, such as fever, to serious complications, such as anemia. A distinctive biological feature of *P. vivax* is the formation of hypnozoites, a latent and undetectable stage in the liver. These hypnozoites can reactivate within up to two years, triggering recurrent infections (relapses) and thus perpetuating the transmission of the parasite. In this context, a robust antibody response directed toward sporozoites, capable of preventing the formation of hypnozoites, represents a promising strategy for relapse prevention. Consequently, the antigens expressed in the pre-erythrocyte stage of *P. vivax* emerge as ideal candidates for the development of vaccines since they interrupt the life cycle of the parasite before the establishment of blood infection and the formation of hypnozoites^5,6^.

Strategies for the development of the malaria vaccine aim to modulate the immune response in different phases of the *Plasmodium* parasite life cycle, which are classically categorized into pre-erythrocyte, blood-stage, and transmission-blocking vaccines. The approach directed to the pre-erythrocyte stage has emerged as the most promising for the development of an effective vaccine, currently representing the main immunization strategy for *P. falciparum* ^3^. Antigens expressed during this phase are considered particularly advantageous targets, since sporozoites infect a limited number of hepatocytes. Additionally, disruption of the parasitic cycle at this stage prevents the establishment of blood infection and, accordingly, the manifestation of the clinical symptoms of malaria, which arise during the erythrocytic phase of the parasite.

Early attempts to develop malaria vaccines employed *Plasmodium berghei* sporozoites attenuated by radiation exposure. Pioneering studies demonstrated that mice immunized with these irradiated *P. berghei* sporozoites showed a significant reduction in hepatocyte infection levels when challenged with non-irradiated sporozoites^7^. This fundamental investigation into the immune response induced by attenuated sporozoites not only provided early evidence of the protective potential of pre-erythrocytic immunization, but also led to the identification of the native circumsporozoite protein (CSP) of *Plasmodium knowlesi* ^8^.

CSP is expressed uniformly and at a high density on the surface of sporozoites of several *Plasmodium* species and plays a crucial role in the invasion of hepatocytes by the parasite^9^. Additionally, CSP shows high immunogenicity in human hosts, making it a promising target for vaccine interventions. Because of these characteristics, the CSP protein emerged as the first antigen extensively explored for the formulation of malaria vaccines. The ability of anti-CSP antibodies to block the invasion of sporozoites into hepatocytes made it the main candidate for the development of effective pre-erythrocytic vaccines against the disease^10-12^.

Regarding *Plasmodium vivax*, the CSP antigen began to be investigated as a potential vaccine target in 1987, with the pioneering study by Barr et *al*.^13^. These researchers demonstrated that immunization of mice with *P. vivax* CSP (*Pv*CSP) induced the production of antibodies capable of efficiently inhibiting sporozoite invasion of hepatocytes *in vitro* assays. Since then, *Pv*CSP became a promising vaccine candidate, with subsequent studies corroborating its ability to induce an immune response that results in a significant reduction in the rate of parasitic infection^13,14^.

Contemporary vaccine formulations such as VIVAX-1^15^, long synthetic peptides (LSP)^16^, and *vivax* malaria protein 1 (VMP 001)^17^ have shown promising results in preliminary studies. However, further research into and development of vaccine formulations with improved efficacy against *P. vivax* is still needed. Given the significant potential that is inherent to the development of a future vaccine for malaria caused by this species, this review aims to perform a detailed analysis of the structure of the CSP protein of *P. vivax* (*Pv*CSP) and to show a comprehensive survey of the studies that investigate the association between the immunogenicity of this antigen in various vaccine formulations and the induction of a protective immune response, both in animal models and in clinical trials with humans.

### Structure of P. Vivax CSP

The *Pv*CSP protein in mature sporozoites has a molecular mass that varies between 45 and 51 kDa, and its expression is conducted in the micronemes of sporozoites, while the parasite is found in the salivary glands of anopheline vectors^18^. The CSP has a sequence that is composed of approximately 373 aa, including a central region with 19 tandem repeats of Asp-Arg-Ala-Asp/Ala-Gly-Gln-Pro-Ala-Gly nanopeptides^19^. Regarding its structure, *Pv*CSP reveals similarities with the CSP proteins of *P. knowlesi* and *P. falciparum*, suggesting a possible conserved function, and it shows a basic structure that consist of three distinct regions in different *Plasmodium* species^20^ *.*

**Figure 1:**
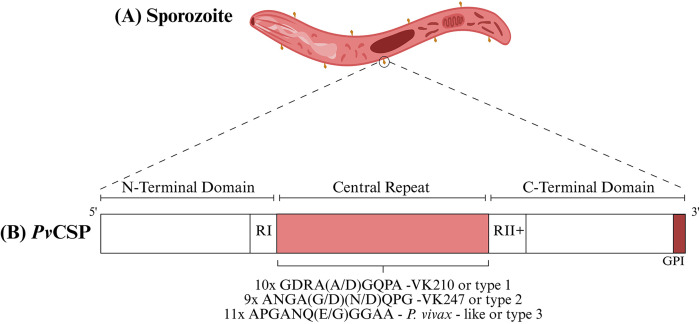
*Plasmodium vivax* circunsporozoite surface protein (*Pv*CSP) structure diagram. *Plasmodium vivax* circumsporozoite and its allelic variants. Schematic representation of the sporozoite of *P. vivax* (A). The CSP sequence in this complex has a central repeat region, containing specific amino acids for *P. vivax* CSP between two non-repeating regions (RI and RII+) at the N-terminal and C-terminal ends of the protein, in addition to a glycosylphosphatidylinositol anchor (GPI) (B).

The central repetitive region of the circumsporozoite protein (CSP) contains immunodominant B-cell epitopes, thus making it a promising target for vaccine development^21^. This region lies between two conserved domains and includes a glycosylphosphatidylinositol (GPI) anchor at the C-terminal.^19^ Additionally, CSP shows species-specific amino acid repeats that are flanked by non-repetitive regions at both the N- and C-terminal. The C-terminal region also features a thrombospondin type-1 domain (TSR) with conserved cysteine residues^19,22^ (Figure 1).

The protein adopts an adhesive conformation when the C-terminal domain of CSP is exposed since the region known as II-plus specifically binds to sulfated glycoconjugates, such as heparan sulfate proteoglycan (HSPG) receptors located in the basolateral domain of the hepatocyte plasma membrane^23,24^. When this domain is masked by the N-terminal region, CSP assumes a non-adhesive conformation, enabling the parasite to migrate through host tissues until reaching target cells. Thus, the structural conformation of CSP plays a key role in enabling the parasite to reach the salivary glands of the mosquito and subsequently invade the liver cells of the host^22^.

*Pv*CSP has three known allelic variants distributed worldwide: VK210, VK247, and *P. vivax*-like. They differ in their nanopeptide sequences in their central repeat regions^25,26^ The VK210 and VK247 alleles are the most common, and show two main types of nanopeptides: GDRA(D/A)GQPA and ANGAGNQPG, respectively. Nonetheless, the *P. vivax*-like variant shows the sequence APGANQ(E/G)GAA, which is different to the other CSP genes of human malaria, but is identical to the CSP of *P. simiovale*, which causes malaria in monkeys^19,27-30^. The region before the repeats contains the sequence KLKQPA (RI), while the region after the repeats (RII) contains the sequence GGNAGG up to EWTPCSVTCGVGVRVRRRVNAA, both are epitopes of B and T cells and are candidates for vaccines^31^. The linear epitopes investigated outside the *Pv*CSP repeat region are not involved in the *in vitro* inhibition of VK247 sporozoites of *P. vivax* ^32^ (Figure 1).

Table 1 summarizes the main findings of these preclinical studies using animal models for *Pv*CSP-based vaccines, which shows the vaccine formulations, animal species used, adjuvants, immunization protocols, and outcomes regarding immunogenicity and protection.

**Table 1:** Summary of preclinical studies evaluating *Pv*CSP-based vaccine candidates in animal models.

Vaccine type and antigen	Animal model	Adjuvant	Dose	Route	Schedule	Outcomes	Article
Protein subunit (purified *Pv*CSP)	BALB/c mice	Alum	50 –250 µg	IM	3 doses	Anti-CSP antibodies; recognition of native CSP; inhibition of sporozoite invasion *in vitro*	Barr *et al*.^13^
Recombinant protein VIVAX-1, NS1-V20	*Saimiri* boliviensis (NHP)	Alum	1 mg	IM	3 doses	High IgG; low/no parasitemia in antigen groups; no added protection in sporozoite group	Collins *et al.* ^15^
MAC (*Pv*CSP repeat + tetanus toxin epitope)	*Saimiri boliviensis* (NHP)	P1005 or RaLPS + P1005 or MF-75 or Alum or PBS	250 µg	IM	3 doses	High IgG; partial/full protection; adjuvant-dependent efficacy	Yang *et al*.^14^
MAPs VI (p11+p25) or VII (p11+tt30)	*Aotus lemurinus* (NHP)	Alum	100 µg	SC	3 doses + booster	High IgG titers; MAP VI for endemic; MAP VII for travellers	Herrera *et al*.^33^
Synthetic peptides (N-terminal, central, C-terminal CSP)	BALB/c mice or *Aotus* (NHP)	Montanide ISA 720	100 µg	IM	3 doses	Immunogenic in both species	Arévalo-Herrera *et al.* ^34^
Recombinant protein (His_6_FliC-*Pv*CS-VK210)	C57BL/6 and TLR5 KO mice	None (FliC as self-adjuvant)	5–10 µg per dose	SC	3 doses	High IgG in WT; low in TLR5 KO; TLR5-dependent immunity	Camacho *et al.* ^35^
Recombinant protein (VMP001: VK210 + VK247)	*Aotus nancymaae* (NHP)	Montanide ISA 720 + TLR9 agonist (CpG10104)	15 or 50 µg	IM	3 doses	Anti-repeat antibodies protective; dose-independent response	Yadava *et al*.^36^
Chimeric protein (y*Pv*CSP-All_FL_ or y*Pv*CSP-All_CT_)	C57BL/6 mice	Montanide ISA 720 or Poly (I:C)	10 μg	SC	3 doses	Strong IgG; recognition of native CSP; delay in parasitemia; no sterile protection	Camargo *et al*.^37^
Chimeric protein (NLP-CSP_CT_ or NLP-CSPR, CSP + mumps N protein)	C57BL/6 mice	Poly (I:C)	10 μg	SC	3 doses	High IgG; 30% sterile protection (VK210); reduced parasitemia	Marques *et al*.^26^
Chimeric protein (NLP-CSP_CT_ or NLP-CSPR)	C57BL/6 mice	Montanide ISA 720 or Poly (I:C)	10 μg	SC	3 doses	Montanide > Poly (I:C) in IgG response; delayed parasitemia; no sterile protection	Gimenez *et al.* ^30^

IM = intramuscular; SC = subcutaneous; MAP = multiple antigenic peptide; MAC = multiple antigen construct; MAPs = Multiple Antigen Peptides; FL = full-length; CT = C-terminal; KO = knockout; Poly(I:C) = polyinosinic:polycytidylic acid; *Pb/Pv* = *P. berghei* expressing *Pv*CSP; NHP = non-human primate; *Pv = Plasmodium vivax*; CSP = circumsporozoite protein; R = repeats; His6 = Histidine tag (6×His).

### Animal model studies

In 1987, Barr *et al*.^13^ published one of the first tests using *P. vivax* CSP as a vaccine antigen for malaria. For this study, three immunizations were performed in mice, with concentrations of 50, 100, or 250 µg of purified protein adsorbed on aluminum hydroxide, and the vaccine formulation was injected intramuscularly. Three weeks after the beginning of the immunizations, the animals already had antibodies against the study protein in the sera that was collected. The antibodies were also tested for the recognition of native CSP, which could inhibit the invasion of sporozoites into hepatocytes *in vitro*.

In 1989, Collins *et al.* ^15^ tested the immunogenic capacity of *P. vivax* CSP in monkeys (*Saimiri sciureus boliviensis*). They applied the CSP protein obtained recombinantly in *Escherichia coli*, another recombinant CSP protein produced in yeast; and, in a third group, they applied irradiated sporozoites. High levels of specific antibodies were observed in the groups that the antigen was administered with an alum adjuvant. Animals immunized with sporozoites had higher antibody titers, but without increased protection after challenge. Animals immunized with the antigens had low or no parasitemia. This was the first study to test the CSP vaccine candidate in challenged monkeys, concluding that the antigen is immunogenic and did not cause toxic side effects^15^.

Eight years after the publication of Collins *et al*.^15^, Yang *et al*.^14^ reported the use of a multiple antigen construct (MAC) containing the *P. vivax* CSP repeat region and tetanus toxin T-helper epitope in different adjuvant formulations to immunize *Saimiri boliviensis boliviensis* monkeys. The animals were separated into five groups, which were three times immunized intramuscularly with MAC with different adjuvants and then were challenged to analyze the protective capacity of the antibodies generated. Results showed that the construction based on the CSP repeat region plus the T-helper epitope of tetanus toxin was highly immunogenic, since the animals produced high levels of antibodies against the CSP repeat region and against sporozoites. Some of the immunized animals were completely protected when exposed to sporozoites, while others showed partial protection. Moreover, the response was strongly influenced by the adjuvant used. Thus, the authors concluded that the MAC vaccine formulation and adjuvant P1005 was able to protect monkeys against infection by sporozoites.

Also in 1997, Herrera *et al*.^33^ reported assays with multiple antigenic peptide (MAP) constructs of the CSP protein, aiming to identify antigenic epitopes that could be used in new MAP constructs for a vaccine against human malaria caused by *P. vivax*. The constructs were tested in *Aotus lemurinus* monkeys, which were immunized subcutaneously with 100 µg of each MAP adsorbed with aluminum hydroxide. Three immunizations and a booster (after five months) were performed. After this, it was observed that MAPs VI (containing p11 and p25 peptides) and VII (based on p11 and p-tt30 peptides) could produce high titers of antibodies against the designed constructs since the first immunization, which increased when booster doses were applied. MAP VI may be a suitable candidate for malaria endemic areas, while MAP VII containing the TT epitope may be a strong candidate for non-immune people visiting endemic regions. The authors thus concluded that a vaccine combining B-cell epitopes derived from the two *P. vivax* CSP variants and/or other epitopes could be used in novel MAP constructs as a strong vaccine candidate for malaria^33^.

In 2011, Arévalo-Herrera *et al*.^34^ published a study in which three synthetic peptides derived from CSP were analyzed. The first synthetic peptide belonged to the amino-terminal region of the CSP antigen, the second one to the central repeat region; and the third to the carboxy-terminal region. Immunizations in Balb/C mice and in *Aotus lemurinus griseimembra* monkeys were performed with definition of similar groups between the two animal models. Group “A” was inoculated with the combination of peptides emulsified in Montanide ISA 720 and Group “B” with peptides emulsified in Montanide ISA 51. The control groups were inoculated with a saline solution emulsified with the two previously mentioned adjuvants. Mice were immunized three times, three weeks apart, while monkeys were immunized three times, with one month between each dose. In summary, the combination of three peptides showed immunogenicity in both animal models. Moreover, the formulation using adjuvant Montanide ISA 51 showed superior results when compared to Montanide ISA 720, but the differences were not statistically relevant.

In a study by Camacho *et al*.^35^, the immunogenicity of an immunodominant region of *P. vivax* CSP (VK210) fused to the FliC protein (*Salmonella enterica* serovar Typhimurium flagellin) and a 6xHIS tail, named His6FliC-*Pv*CS-VK210, was investigated. For the antigen immunogenicity tests, female C57BL/6 (H-2 b) and TLR5 (KO) deficient mice were immunized three times subcutaneously, with 21 days between each dose. A significant serum anti-*Pv*CSP specific IgG response persisted for up to nine weeks after the third dose. Moreover, the activation pathway of the immune response against this antigen occurs via TLR5, since TLR5 (KO) mice developed low antibody titers when inoculated with His6FliC-*Pv*CS-VK210. Therefore, the authors concluded that FliC can be used as an adjuvant and antigen carrier for malaria.

Yadava *et al.* ^36^ reported the immunogenicity of the vaccine antigen named VMP001, which is based on the sequences VK210 and VK247 of *Pv*CSP. Two TLR4 agonists were used as adjuvants. *Aotus nancymaae* and *Aotus lemuirinus grisiemembra* monkeys were immunized in groups that received 15 µg or 50 µg of VMP001 per dose, and a control group immunized with 50 µg of *P. falciparum* LSA-1. Both groups immunized with low and high doses showed similar responses after the second dose; however, only the anti-repeat antibodies could generate protection in the challenged animals. Thus, the study served as the basis for choosing the next vaccine formulations for malaria caused by *P. vivax*.

In 2018, Camargo *et al*.^37^ evaluated the humoral response and protective capacity of antibodies generated against two chimeric proteins: y*Pv*CSP-All _FL_, which included fused repeat regions flanked by the N- and C-terminal domains (Full-Length, FL), and yPvCSP-All_CT_, comprising the fused repeat regions, the C-terminal domain (CT) and the RI region (C-Terminal, CT), both administered together with Montanide ISA720 or Poly (I:C) adjuvants, including sequences from all three alleles (VK210, VK247 and *P. vivax*-like). A robust, specific humoral response was observed, with no relevant difference between the proteins, nor between the adjuvants used. Moreover, the antibodies generated against both proteins recognized the native CSP protein of *P. vivax*. After a challenge infection of the inoculated mice, a delay occurred in the time required to reach 1% parasitemia, in both immunization regimens. However, these failed to fully protect the animals against the disease. The authors concluded that the tested vaccine formulations can induce immunity and partial protection against the two major *Pv*CSP alleles evaluated (VK210 and VK247), thus becoming the basis for further research aimed at developing a vaccine for *P. vivax*.

In a study reported by Marques *et al*.^26^, the alleles VK210, VK247, and the *P. vivax*-like region were all fused to the nucleocapsid protein of the mumps virus, with and without the conserved C-terminal region (NLP-CSP_CT_ and NLP-CSP_R_, respectively). The two formulations were tested in mice, which were subcutaneously inoculated with recombinant proteins together with adjuvant Poly(I:C) HMW (high molecular weight). Subsequently, the group immunized with NLP-CSP_CT_ was challenged. In this study, the two formulations elicited high antibody titers for the three repeated regions of *Pv*CSP, with the *P. vivax*-like region generating the most specific antibodies. Moreover, the NLP-CSP_R_ formulation predominantly induced the IgG2c subclass, in contrast to NLP-CSP_CT_, which showed predominance of IgG1. The immunized animals were challenged with a sporozoite strain from *Plasmodium berghei* ANKA, which expresses CSP VK210 repeats of *P. vivax* (*Pb*/*Pv*VK210). The antibodies generated against the different antigens tested conferred sterile protection in approximately 30% of the animals, in addition to conferring a significant decrease in parasitemia levels of immunized mice, with efficacy restricted to the allelic variant VK210.

In 2021, Gimenez *et al*.^30^ reproduced the assays described by Marques *et al*.^26^ and also evaluated the efficacy of immunizations with the use of the adjuvant Montanide ISA720. Moreover, the mice were challenged with chimeric parasites that express *Pv*CSP-VK210, *Pv*CSP-VK247 and a new strain of *P. berguei* that expresses the third allelic variant *Pv*CSP-like, thus making it possible to analyze the protective capacity against the three allelic regions. There was a significant difference in the production of specific antibodies in the groups immunized with the proteins combined with the Poly (I:C) adjuvant and in the groups immunized with the protein combined with Montanide ISA 720, the latter being responsible for higher specific IgG titers. Induction of sterile protection by the studied vaccine formulations could not be observed in the challenge trials with chimeric sporozoites, only a significant delay to reach 1% parasitemia. The authors concluded that the adjuvant Montanide ISA 720 had a superior performance in relation to humoral response and protection. However, the formulation showed limitations regarding its safety, due to its high reactogenicity. Moreover, due to the lack of protection provided by the formulations tested, they concluded that the mechanisms involving protection must be analyzed so that they can proceed with clinical trials.

### Clinical studies

In 1990, Gordon *et al*.^38^ published a clinical study for a vaccine against *P. vivax* malaria based on an immunizer consisting of a fragment of 20 tandem repeats of CSP (V20) fused with a non-structural protein of influenza (NS1). The study evaluated the safety and immunogenicity of the vaccine candidate named NS1V20, which was adsorbed on alum. Thus, 13 volunteers who had never contracted malaria were immunized with 10, 100, or 1,000 µg of NS1V20. After the first immunization, two more boosters were performed in each group, and six of the nine volunteers who received the dose of 100 or 1,000 µg developed antibodies specific to the CSP region used after the first dose. As for the boosters, they were not successful in continuing to trigger a response against the vaccine, so the antibody levels decreased over time. The authors concluded that the NS1V20 immunogen was safe and well tolerated for use in humans at high concentrations up to 1,000 µg. Nonetheless, the authors hypothesize the existence of immunosuppressive regions in the repeat epitopes of the CSP protein, suggesting that the development of a vaccine for *P. vivax* should be based only on immunodominant regions of the CSP^38^.

In a safety and immunogenicity study by Herrington *et al*.^39^, 30 volunteers received intramuscular doses of 50, 100, 200, or 400 µg of *Pv*CSP, a recombinant protein corresponding to 70% of the native protein and adsorbed on alum. The vaccine was well tolerated, and IgG antibodies against *P. vivax* CSP were detected by Western blot in all the volunteers who received the 400 µg dose and in six individuals from the 200 µg group. However, when antibody responses were further evaluated using indirect immunofluorescence assays with sporozoites or enzyme-linked immunosorbent assay (ELISA) against CSP, only low levels of reactivity were observed.

Herrera *et al*.^40^ evaluated the safety and immunogenicity of vaccines composed of synthetic peptides derived from the *Pv*CSP protein. Three peptides were used, named N (amino acids 20 to 96 of *Pv*CSP), R (VK210) or C (amino acids 301 to 372 of *Pv*CSP). In all, 69 volunteers, who had never had contact with malaria, received three applications of each vaccine formulation in immunizations of 10, 30 or 100 µg/dose. The peptides were formulated with the Montanide ISA 720 adjuvant. After immunizations, peptide N at a dose of 100 µg incited the highest level of antibodies. Regarding the recognition of *P. vivax* sporozoites by these antibodies, all three peptides induced the production of antibodies that could recognize the native protein in the immunofluorescence test. Moreover, peripheral blood mononuclear cells of most of the immunized volunteers produced interferon-gamma in *in vitro* assays. With this study, the authors concluded that the tested vaccines were safe, well tolerated by humans, and immunogenic, however, it is necessary to optimize these to progress to the phase II clinical trial^40^.

Arévalo-Herrera *et al*.^41^ continued the studies published by Herrera *et al*.^40^, and proceeded to phase I clinical trials, this time associating the tested peptides with two other adjuvants: Montanide ISA 720 and Montanide ISA 51. Three doses of the vaccine containing peptide mixtures in concentrations of 50 or 100 µg of each peptide were administered. As to the results, all the formulations proved to be immunogenic, inducing specific antibodies and production and IFN-γ, with about 96% of volunteers producing IgG antibodies that were specific for all the peptides tested after the third immunization with the peptide mixture. Furthermore, of all the sequences tested, peptide N caused the highest IgG titer when administered at a dose of 300 µg, together with the adjuvant Montanide ISA 720. The adjuvant Montanide ISA 51 induced higher titers of antibodies that could recognize the native protein when compared to Montanide ISA 720. In conclusion, the study demonstrated that the combination of a long synthetic peptide derived from *Pv*CSP formulated with both Montanide ISA 720 and 51 is safe, well tolerated, and immunogenic. Moreover, peptides formulated with Montanide ISA 51 induced a better response of recognition antibodies to native sporozoite CSP, demonstrating that the adjuvant used in the vaccine formulation is an important point to be further analyzed^42^.

Arévalo-Herrera *et al*.^43^ continued their studies in a clinical trial of the vaccine formulation *Pv*CSP LSP adsorbed on Montanide ISA-51 in malaria-naive subjects (phase IIa) and in semi-immune volunteers (phase IIb). Immunizations were performed on 35 volunteers divided into two groups: the first group corresponding to healthy subjects, without treatment for malaria and semi-immune, who received three doses of the vaccine; and the second corresponding to the placebo group. The immunization regimen was conducted at intervals of zero, two and six months. The vaccine is safe and well tolerated, and, by using ELISA, it was found that there was seroconversion of all the volunteers in the experimental group, both “naive” for malaria and semi-immune, after the first immunization. However, reactivity was lower in the semi-immune group despite previous exposure to the parasite. There was a small but significant increase in the production of antibodies against the three protein fragments in some volunteers after the third immunization, again being greater in the “naive” group than in the semi-immune. There was a drastic reduction in parasitemia after a challenge infection in phase IIa (54%) and phase IIb (64%) volunteers of the “naive” group. The authors concluded that significant protection was observed in both groups of volunteers, demonstrating the importance of developing the *Pv*CSP-based vaccine. They hypothesized that pre-exposure to *P. vivax* malaria in endemic areas influenced vaccine-induced immunity. As a perspective, the group will seek to conduct comparative trials with larger groups of volunteers^43^.

Bennett *et al*.^44^ conducted the first phase I clinical trial in humans, using the formulation VMP001/AS01, which tested the reactivity, immunogenicity, and efficacy of the challenge infection with *P. vivax* sporozoites in healthy “naive” adult volunteers. Thirty volunteers were divided into three cohorts and received three doses of the immunogen at concentrations of 15 µg, 30 µg, and 60 µg/dose. Fourteen days after the third immunization, a controlled human malaria infection (CHMI) was performed. After the immunizations, it was observed that the vaccine was well tolerated in the three applications. However, the effectiveness of the vaccine was 0%, despite it triggering a significant delay in the average pre-patent period. Regarding antibody titers, they observed that there was a 5- to 8-fold decrease six months after the CHMI, when compared with the titers presented before the challenge. These titers, in turn, remained higher than those detected after the first immunization. In conclusion, although the vaccine was not able to protect any of the subjects tested, the authors observed that a significant proportion of the subjects had a delay in infection, which was related to the antibodies from the repeated region of the antigen used in the vaccine^44^.

Table 2 summarizes the main findings of these clinical studies on *Pv*CSP-based vaccines, and compiles key information regarding vaccine formulations, dosages, adjuvants, immunogenicity, and safety outcomes.

**Table 2:** Summary of clinical trials evaluating *Pv*CSP-based vaccine candidates in humans.

Vaccine type and antigen	Study population	Adjuvant	Dose	Route	Schedule	Outcomes	Article
Fusion protein (NS1V20: influenza NS1 + 20 CSP repeats)	13 malaria-naive adults	Alum	10, 100, 1,000 µg	Not reported	3 doses	Safe and well tolerated; antibodies detected after 1st dose (100–1,000 µg); decline after boosters	Gordon *et al.* ^38^
Recombinant *Pv*CSP (70% of native protein)	30 malaria-naive adults	Alum	50, 100, 200, 400 µg	IM	3 doses	Safe; IgG in 400 µg group by Western blot; low reactivity by IFA and ELISA	Herrington *et al.* ^39^
Synthetic peptides (N, R [VK210], C fragments)	69 malaria-naive adults	Montanide ISA 720	10, 30, or 100 μg/dose (per peptide)	IM	3 doses	Safe and immunogenic; peptide N (100 µg) induced highest IgG and IFN-γ; all peptides recognized native CSP	Herrera *et al*.^40^
Long synthetic peptide (*Pv*CSP-LSP)	21 adults	Montanide ISA 720	100 μg per peptide	IM	3 doses	Safe; all naive seroconverted; semi-immune = lower response; reduced parasitemia post-challenge (54–64%)	Herrera *et al.* ^41^
Chimeric recombinant protein (VMP001: VK210 + VK247)	36 healthy, malaria-naive adults	AS01B (MPL + QS-21 liposomal formulation)	15, 30, 60 µg	IM	3 doses	Safe; no sterile protection; delayed pre-patent period; antibody titers dropped 5–8× in 6 months	Bennett *et al*.^44^
Synthetic peptide mixtures (N, R, C fragments)	35 volunteers (17 naive - Phase IIa; 18 semi-immune - Phase IIb)	Montanide ISA 51	50 μg (first dose: N+C only) 300 μg (boosts: N+R+C)	IM	3 doses	96% IgG seroconversion; peptide N (300 µg) + ISA 720 = highest IgG; ISA 51 = better recognition of native CSP	Arévalo-Herrera *et al*.^42^

NS1 = Non-structural protein 1 (influenza virus); CSP = Circumsporozoite protein (*Plasmodium vivax*); LSP = Long synthetic peptide; IM = Intramuscular; SC = Subcutaneous; CHMI = Controlled human malaria infection;; N = Amino-terminal fragment of CSP; R = Repeat region of CSP (VK210 variant); C = Carboxy-terminal fragment of CSP; *Pv*CSP-LSP = *Plasmodium vivax* Circumsporozoite Protein Long Synthetic Peptide; VMP001 = Chimeric recombinant protein combining VK210 and VK247 variants of CSP; AS01B = Liposomal adjuvant formulation containing MPL (monophosphoryl lipid A) and QS-21.

## CONCLUSION

The development of an effective vaccine against *P. vivax* requires a careful analysis of multiple factors. The concentration of the administered immunogen emerges as a critical parameter, with evidence suggesting its influence on the results of the immune response. Additionally, the choice of adjuvant represents an aspect of paramount importance: it must have an adequate tolerability and safety profile, while being able to potentiate the induction of a robust and highly specific humoral response against the *Pv*CSP antigen.

*P. vivax* malaria vaccines based on the *Pv*CSP protein consistently demonstrate immunogenicity and the ability to confer partial protection in animal models, evidenced by a significant delay in the establishment of parasitemia after challenge. However, translating these promising results into clinical trials has been challenging. Although the formulations tested in humans have been shown to be well tolerated and capable of inducing the production of specific antibodies against *Pv*CSP antigen in all the approaches evaluated, the clinically relevant protective immune response has not yet been achieved. Notably, the antibodies generated demonstrated recognition of the native protein on the surface of sporozoites *in vitro*, and in line with preclinical findings, some formulations were able to induce a significant delay in parasitemia in the volunteers, despite not preventing infection.

New formulation and administration strategies are being explored and successfully applied in vaccines against *P. falciparum* to overcome the limitations. The incorporation of innovative adjuvants, such as *Bacillus subtilis* spores bioengineered to present the *Pf*CSP protein on its surface, the Matrix-M adjuvant, based on saponin, used in recent studies against *P. falciparum*, DNA, or RNA formulations, and liposomal delivery systems, among others, represent promising advances. Additionally, the investigation of alternative immunization routes, such as mucosal (oral and nasal) routes, aims to improve the availability and efficacy of vaccines against malaria caused by *P. falciparum*. Adaptation and application of these innovative strategies to the development of *Pv*CSP-based vaccines for *P. vivax* are crucial to achieve robust and long-lasting protection against this important cause of global morbidity.

## Data Availability

The complete anonymized dataset supporting the findings of this study is included within the article itself.
